# Aptamer Applications in Neuroscience

**DOI:** 10.3390/ph14121260

**Published:** 2021-12-03

**Authors:** Meric Ozturk, Marit Nilsen-Hamilton, Muslum Ilgu

**Affiliations:** 1 Roy J. Carver Department of Biochemistry, Biophysics and Molecular Biology, Iowa State University, Ames, IA 50011, USA; ozturk@iastate.edu (M.O.); marit@iastate.edu (M.N.-H.); 2 Department of Biological Sciences, Middle East Technical University, Ankara 06800, Turkey; 3 Ames Laboratory, US DOE (United States Department of Energy), Ames, IA 50011, USA; 4 Aptalogic Inc., Ames, IA 50014, USA

**Keywords:** aptamer, neuroscience, neurological diseases, neurological disorders, neurotoxins, cancer

## Abstract

Being the predominant cause of disability, neurological diseases have received much attention from the global health community. Over a billion people suffer from one of the following neurological disorders: dementia, epilepsy, stroke, migraine, meningitis, Alzheimer’s disease, Parkinson’s disease, multiple sclerosis, amyotrophic lateral sclerosis, Huntington’s disease, prion disease, or brain tumors. The diagnosis and treatment options are limited for many of these diseases. Aptamers, being small and non-immunogenic nucleic acid molecules that are easy to chemically modify, offer potential diagnostic and theragnostic applications to meet these needs. This review covers pioneering studies in applying aptamers, which shows promise for future diagnostics and treatments of neurological disorders that pose increasingly dire worldwide health challenges.

## 1. Introduction

Aptamers are single-stranded DNA, RNA, or synthetic XNA molecules that can fold into unique three-dimensional (3D) structures by which they bind their target molecules with high specificity and affinity [1,2]. An approach to selecting aptamers was achieved in 1990 by three separate groups [3,4,5]. The method is now known as the Systematic Evolution of Ligands by Exponential Enrichment (SELEX). Since then, many aptamers have been selected for use in areas of basic science and an increasing number of aptamers are under development for applications in therapeutics, diagnostics, and imaging [6,7,8,9,10].

The SELEX method simulates Darwinian evolution in vitro. It includes a number of selection rounds, and, after each round, exponential amplification of the “fittest” oligonucleotides (oligos) is carried out. One round of traditional SELEX includes three steps in the order (1) incubate the desired target molecule with a pool of oligos with a central 20–60 nucleotide region of randomized sequence surrounded by terminal regions of constant sequence that will be templates for later polymerase chain reactions (PCRs), (2) capture the oligos in the pool that successfully bind the target, and (3) amplify the captured oligos by either PCR or reverse transcription-PCR (rt-PCR), depending on the type of oligo, DNA or RNA respectively. Rounds of counter-selection are also included, in which the nucleic acid pool is passed over an empty supporting matrix, without the desired target or over the supporting matrix with one or more other molecules that might be structurally related to the target molecule. In counter selection, the oligos that do not bind the supporting matrix, or the structurally related molecules are captured and continued through more rounds of SELEX. Counter selection is performed for many purposes, such as to eliminate oligos that interact with (1) the supporting matrix, (2) analogs or molecules that are structurally related to the target, or (3) molecules present at high concentrations in the biological matrix (such as serum or tissue extract) in which the selected aptamer will function. The main goal of counter selection is to increase aptamer specificity for the desired target over other potential molecular competitors. Besides conventional SELEX, recently developed structure-switching SELEX is becoming popular [11,12,13]. In this approach, oligos are required to change their structures when they interact with the target, in order to dissociate from a complementary capture sequence. This ensures that the selected aptamers change in structure upon ligand binding. Other alternative SELEX methods have been developed including CE-SELEX, Cell-SELEX, and in-vivo SELEX, as summarized in Table 1. It is quite possible to combine different SELEX procedures to develop even more complex techniques. After aptamers are selected by SELEX, they are further modified to improve their specificities and affinities by a number of approaches that, in toto, can be referred to as maturation.

There are several considerations in selecting and maturing aptamers. First, the structures of aptamers and aptamer-target interactions are affected by the temperature and the buffer components, including ions, ionic strength, and pH. Thus, one of the most important points in SELEX design is to mimic the environment (biological matrix or salt/ buffer composition) during selection in which the aptamer molecular target is found and the interaction between aptamer and target will occur. Choosing appropriate buffer compositions and incubation temperatures will ensure the optimal performance of the selected aptamers under the conditions in which they will be applied [4,30,31,32]. Second, aptamer stability may need to be improved, as DNA and RNA aptamers are potential targets for nuclease attack. This is especially the case for RNA, because of the 2′OH group, which is used by ribonucleases in an electrophilic attack of the phosphate of the nucleic acid backbone for hydrolysis. Thus, RNA is more labile to high temperatures and pH than DNA. Aptamers can be matured by post-selection chemical modifications to overcome this susceptibility. However, such modifications can result in the aptamer losing specificity and/or affinity. Therefore, it is usually better to include modified nucleotides during SELEX. Many possible aptamer modifications are listed in Table 2.

Aptamers can be selected that selectively bind most viruses, cells, bacteria, proteins, toxins, and peptides with high affinities [45,46,47,48]. They can be used several times without noticeable disruption of activity. In other words, they can be separated from their targets for further use. By contrast, antibodies can only be utilized only a few times before they lose functionality. Nucleic acid aptamers can also be matured to be more stable than antibodies and enzymes, particularly at higher temperatures [2]. Moreover, once the sequence of an aptamer is known, its chemical synthesis and purification to homogeneity are highly reproducible and inexpensive. These properties give aptamers the potential to substitute for antibodies as components of sensing units [49]. The flexibility of aptamers’ structures and their ability to “mask” regions of an aptamer sequence with complementary oligos provides options for signaling that allow the incorporation of aptamers into many sensor platforms, which are not readily adaptable to antibodies. These properties motivate aptamer-based biosensor development [50,51,52].

Developing new tools for diagnosis is one of the most salient areas in the biosensor field, with many investigators seeking to create easy, efficient, and cheaper methods to achieve early diagnosis and precision medicine. The inclusion of aptamers in biosensors (named “aptasensors”) took root in the 1990s. An early example of aptamers as bio-recognition elements is an optical biosensor that used fluorescently labeled aptamers against human neutrophil elastase in a homogenous assay [53]. Since then, many types of aptasensor designs have been reported, most with aptamers on solid supports, and with electrochemical, optical, mechanical, and acoustic signals [45,54,55,56]. Some examples are discussed later in this article.

Aptamers have also been applied in therapeutics. Modifications that result in increased half-lives and improved pharmacokinetics make some aptamers good options for clinical application. Compared with antibodies, aptamers are much less immunogenic, and their actions can be inhibited by reverse complementary oligonucleotides (Munzar et al., 2019). These important characteristics recommend aptamers for development as therapeutics. Macugen (pegaptanib sodium), which selectively recognizes vascular endothelial growth factor (VEGF), was the first aptamer to be approved as a therapeutic agent (Tobin, 2006). Since then, several aptamers have reached different stages of clinical trials in which they are being tested for treating medical conditions like small cell lung cancer, von Willebrand factor-related disorder, angiomas, and renal cell carcinomas [51,57,58,59,60].

Another potential therapeutic application of aptamers involves delivery systems [61,62]. To achieve efficient results in these systems, aptamers should recognize cell surface proteins in their native forms. Cell-SELEX was developed to select against cell-surface proteins that are frequently difficult to purify in their native forms [63]. An aptamer that recognizes a cell surface protein can coopt the receptor to internalize a cargo attached to the aptamer. With this method, one can select aptamers to receptors primarily expressed on a single cell type that can be targeted in vivo [57]. For instance, anti-PSMA (prostate-specific membrane antigen) aptamers were developed for the delivery of siRNAs to tumors in mice or cultured cells [62,64,65,66].

**Figure 1:**
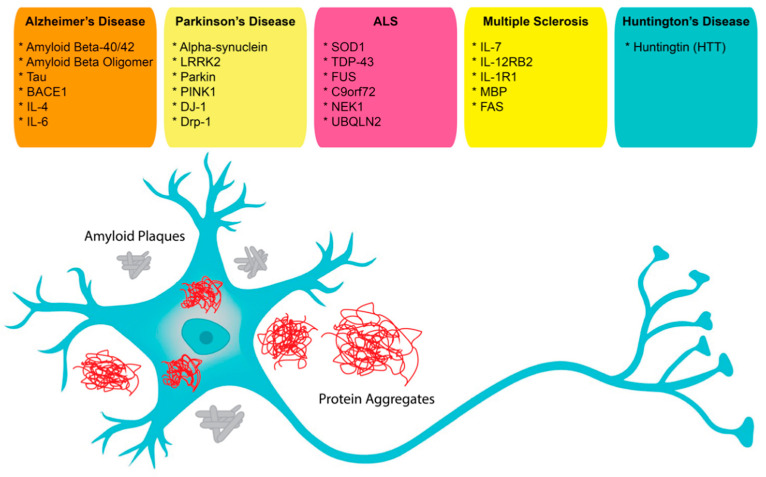
Possible aptamer targets in neuroscience.

In neuroscience, the use of aptamers has, so far, been limited, albeit while having a very wide range of potential applications. Aptamers have been reported as possible treatment options for neurodegenerative diseases that result in the loss of central nervous system (CNS) structure and function, including Alzheimer’s disease (AD), Parkinson’s disease (PD), Creutzfeldt–Jakob disease, motor neuron diseases, and Huntington’s disease (HD) (Figure 1). Currently, around 50 million people in the world face dementia, for which the global healthcare cost is close to a trillion US dollars per year. This makes dementias one of the biggest medical and economic problems for our society [67]. As well, according to the United Nations and the World Health Organization, the world population over 65 years old is estimated to double by 2050. Therefore, age-dependent neurodegenerative diseases will require increasing expenditures for diagnosis and treatment [67]. These critical demands for methods to facilitate early diagnosis and new therapeutic applications could be met by aptamers, which could also be solutions for some of the underlying complications [68,69].

Aptamers are being developed for applications in brain imaging technologies (MRI, PET, etc.), neurotransmitter visualization, diagnosis, and therapeutics for brain tumors and other brain-related diseases. In many of these approaches, aptamers are labeled with radiotracers, such as fluorine-18 for PET imaging [70]. In this review, we will discuss the achievements and challenges of applying aptamers to brain imaging and neural diseases.

## 2. Molecular Detection

### 2.1. Neurotoxin Detection

Neurotoxins are a chemically diverse group of pharmacologically active compounds. They exert clear biological effects on the nervous systems of organisms but differ in their chemical structures and mechanisms of action. They change action potentials, and so interfere with the transmission of nerve impulses. To properly assess their risks to human health and environmental impacts, it is essential to use sufficiently sensitive techniques for their accurate identification. Nucleic acid aptamers have been utilized to validate the presence of these toxins in water and biological samples with high sensitivity and selectivity [71]. Studies have been reported that demonstrate the accurate identification of botulinum neurotoxins (Botulinum A, E), saxitoxin, brevotoxin, paraquat, and tetrodotoxin by aptasensors.

Botulinum neurotoxins (BoNTs) are among the deadliest neurotoxins and could potentially be employed as bioterrorism agents. To address the crucial need for a sensitive, effective, and real-time detection system for BoNTs, a sol–gel-based SELEX was used to isolate DNA aptamers against botulinum neurotoxin type E (BoNT-E) [47]. The aptamer candidate sequences, after the fifth round, were analyzed by next-generation sequencing (NGS) and high-frequency aptamers were characterized. In these aptamer families, aptamer BT5.6 showed a high affinity, of 53 nM Kd, for BoNT-E and was immobilized on a graphene oxide platform. This sensor displayed a 0.83 nM limit of detection (LoD) for BoNT-E.

Brevotoxins (BTXs) and saxitoxin are marine neurotoxins that cause neurological shellfish poisoning (NSP). BTXs are characterized by high toxicity and rapid onset of NSP. Technical difficulties related to their analysis have encouraged researchers to search for alternative, effective, and cheap detection methods for these strong neurotoxins. For this purpose, an electrochemical aptasensor was developed to detect BTX-2, for which the selected DNA aptamers were characterized using electrochemical impedance spectroscopy (EIS) and fluorescence. The aptamer BT10, with an affinity of 42 nM for BTX-2, was incorporated into a label-free competitive impedimetric biosensor that demonstrated a detection limit of 95 pM of BTX-2. This aptasensor was able to detect BTX-2 in a spiked shellfish extract with a reliable response in the presence of shellfish matrix [72]. As the second class of marine neurotoxins, saxitoxin was characterized for public health significance. Novel studies were required to be developed as suitable alternatives because of the technical and ethical issues of currently applied systems. To address this need, a label-free optical biolayer interferometry competitive aptasensor was developed. The biosensor has a detection limit of 1.66 nM for saxitoxin, with a high degree of selectivity and sensitivity, good stability, and rapid detection capability [73].

Similar to marine toxins, neurotoxins released upon snakebite are among the most powerful and lethal toxins. Unfortunately, conventional snakebite diagnostics based on blood coagulation assays and clinical symptoms cannot provide an accurate diagnosis. Also, batch-dependent variations of antibody performance complicate their use in diagnostics. A high-affinity truncated version of an α-*Tox*-FL aptamer, originally selected against α-bungarotoxin of *Bungarus multicinctus* (α-Tox-T2) with a higher affinity (Kd = 2.8 nM) than the parent α-Tox-FL (18 nM), was applied in an enzyme-linked aptamer assay format. In this setup, the α-Tox-T2 aptamer could detect α-toxin in as little as 2 ng of crude venom, which demonstrated its potential for *Bungarus caeruleus* venom detection [74].

### 2.2. Neurotransmitter Detection

To explore the functional complexities of the brain, monitoring neurotransmitter activities and signaling pathways can be used. Neurotransmitters are essential for information transfer between cells and play key roles in neurological disorders such as epilepsy, Parkinson’s disease (PD), and Alzheimer’s disease (AD). In a number of studies, aptamers specific for dopamine, epinephrine, or serotonin have been used to develop aptamer-based biosensors for neurotransmitter detection, in vivo and in vitro [75].

A catecholamine neurotransmitter, dopamine (DA) regulates aspects of human metabolism, cardiovascular, and renal system functions. Several neurodegenerative diseases, especially PD, were shown to be directly correlated with the abnormal metabolism of DA [76]. Thus, developing highly sensitive and selective biosensors for DA determination is important in biological systems. The first RNA aptamer against DA, dopa2, bound DA with an affinity represented by Kd = 2.8 nM [77]. Using this aptamer, a label-free electrochemical aptasensor based on a graphene–polyaniline composite film successfully detected the presence of DA in human serum, with an LoD of 1.98 pM [78]. In a more recent study, another DA aptasensor was developed composed of DNA aptamer as a sensor and a grass carp skin collagen-graphene oxide (GCSC-GO) composite as a transducer. The composite was prepared via ultra-sonication and characterized by infrared (IR) and Raman spectroscopy, atomic force microscopy, and EIS. This system included aptamer immobilization to collagen followed by differential pulse voltammetry. Although the aptasensor demonstrated a higher LoD of 0.75 nM, compared to the earlier sensor with 2 pM, it showed high selectivity for DA over closely related compounds and accurately measured DA in the presence of 10% serum [79].

For the analysis of various molecules with free-label, high sensitivity, and selectivity, a microcantilever array sensor with aptamers was recently reported. The sensor consists of an array of 12 microcantilevers modified with thiolated DA aptamers and uses gold nanoparticles (AuNP) conjugated with a stretch of DNA that is complementary to the DA aptamer on the microcantilever surface. Initially hybridized with the DA aptamer, the AuNP-DNA conjugates are released when the aptamer binds DA, which enhances the surface stress of the array by about 15 times and increases deflection. The system has a linear response in the range from 0.5 to 4 μM of DA with an LoD of 77 nM. Its specificity was demonstrated with 12 structural and functional analogues including L-DOPA, none of which initiated deflections [80].

Another neurotransmitter, serotonin, plays a role in neuromodulation activities affecting sleep, aggression, appetite, and sexual activity. It is produced in different body parts, such as the brain, spinal cord, platelets, and intestine [81,82,83,84]. Imbalances of peripheral serotonin levels have been linked to several disease conditions, including hypertension, kidney disease, and depression. Thus, using serotonin to predict the disease state and to initiate proper treatment has clinical importance. A plasmonic assay for serotonin was developed with an aptamer–AuNP conjugate for which aggregation is altered in the presence of serotonin. The change in particle aggregation can be measured as a shift in the peak wavelength of absorption. The assay showed a specific response to serotonin and gave no statistically significant signal with its metabolites, 5-hydroxyindoleacetic acid (5-HIAA), epinephrine, or norepinephrine when each was tested at 1 µM. Fetal bovine serum, as a mimic of human serum, also initiated no signal [85]. Recently, implantable aptamer–field-effect transistor (FET) neuroprobes have been developed for monitoring serotonin levels in brain tissue. Ultrathin In_2_O_3_ surfaces of nanoscale FETs were modified with aptamers and these neuroprobes enabled fM serotonin detection limits in vivo with minimal biofouling [86]. With additional neuroprobes, spatiotemporal recordings of neural activity will be possible so brain functions can be understood with much deeper level.

Epinephrine is a catecholamine neurotransmitter with a wide range of physiological functions for which abnormal levels may cause health problems like arrhythmia, myocardial infarction, blood pressure increase, and pulmonary edema. It is important to quantify circulating epinephrine for observing these related disease states. With this aim, a colorimetric detection method, based on the interaction of epinephrine with aptamer functionalized Au-NP, was developed. The system is less expensive and more specific than other techniques for quantifying epinephrine, such as by liquid chromatography, and spectrophotometry. Also, a detection limit of 0.9 nM was determined by UV-visible spectroscopy, which is the lowest detection limit recorded for epinephrine using any colorimetric method. Analogs of epinephrine, 3,4-dihydroxyphenylacetic acid (DOPAC), tryptophan, ascorbic acid, dopamine, tyrosine, and homovanillic acid, showed very little or no response, which indicates a high specificity of the aptasensor [87].

A change in the level of a single neurotransmitter can indicate any of several diseases, which makes it highly unlikely that a disease diagnosis can be achieved based on the measurement of a single neurotransmitter. Therefore, the ability of most aptasensors to be integrated into a multiplex platform will result in diagnostic tools better suited to clinics and with the potential of supporting more accurate disease identifications.

### 2.3. Biomarker Detection

A biomarker is any substance, structure, or process in the body that either results in an outcome or indicates the presence of a specific disease. Many biomarkers are substances that can be measured in biofluids such as the blood, saliva, and urine. The main problem of biomarker characterization for neurological diseases is the blood-brain barrier (BBB) through which many molecules, including many proteins, cannot pass. Although proteins are the dominant biomarkers for certain diseases, small peptides, such as neuropeptide Y or fragments of proteins, are more likely to pass through the BBB and be detected in the circulation. Metabolites are another important group of biomarkers for neurological disorders.

NeoVentures Biotechnology Inc. has developed an aptamer selection approach called FRELEX, which allows the selection of aptamers based on competition between a short oligonucleotide attached to a surface and the target. Like for cell-SELEX and other forms of structure-switching SELEX, this protocol can be performed with a sample containing a mixture of components without prior knowledge of the target (s) to which aptamers will be selected. With these forms of SELEX, counter selections are performed with particular subsets of components to drive the selection of aptamers that fulfill the desired specificities. Using FRELEX, aptamers were selected against serum from transgenic mice that overexpressed the human tau protein. To drive aptamer selection towards human tau and the consequences of tau overexpression, serum from wild-type mice was used in the counter selection. The group identified the enriched aptamer sequences using NGS and characterized certain aptamers for late-stage transgenic mice and wild-type mice by comparing their relative abundances. They hypothesized that the difference in abundance reflects the respective concentration of the epitopes (increased in response to tau overexpression) with which the aptamers can interact [29].

## 3. Diagnostic and Therapeutic Applications

Neurodegenerative diseases (NDs) involve the degeneration of neurons in the brain that results in the loss of structures and functions of the central nervous system. ND can be caused by aging, genetic and environmental factors. Dementia is one of the most common age-related NDs as is Alzheimer’s disease (AD). Parkinson’s disease (PD) can be due to genetic mutations and/or environmental toxins. Amyotrophic lateral sclerosis (ALS), Multiple Sclerosis (MS), Huntington’s disease (HD), and prion diseases are other common NDs with genetic links. Unfortunately, neither effective cures nor early detection strategies are available for these diseases. Like the widely used antibodies, aptamers have become attractive agents to apply to developing novel biosensors for early diagnosis of NDs and cure of these diseases [88].

### 3.1. Alzheimer’s Disease

As an age-related progressive brain disorder resulting in mental deficiencies, AD is the most common form of dementia. Its pathology is characterized by the aggregation of amyloid-β (Aβ) derived from the amyloid precursor protein (APP) and initiated in the brain region of the hippocampus. Although scientists hypothesized that Aβ-induced neurotoxicity was correlated with insoluble Aβ plaques (AβP) and fibrils (AβF), recent evidence indicates that soluble Aβ oligomers (AβO) are also associated with AD onset. Therefore, AβO has been identified as an attractive biomarker for early diagnosis of AD, and Aβ and tau are considered as significant therapeutic targets for treating AD [89].

#### 3.1.1. AD Diagnostics

Early described aptamers against AβO had low affinities and specificities, but later studies were more successful in identifying aptamers that selectively recognized fibrils of a 40-residue form of Aβ (Aβ40) but not Aβ40 polymers [90]. However, the aptamers lacked high specificity for Aβ40 fibrils over fibrils of several other amyloidogenic proteins that were tested. A DNA aptamer selected to interact with α-synuclein (α-syn) oligomer with 68-nM affinity was also shown to bind AβO with 25-nM affinity [91]. In 2019, using the same aptamers obtained by Tsukakoshi et al., a label-free electrochemical aptasensor was developed for more specific recognition of AβO [92]. The aptamer self-assembles on gold rod electrodes via thiol (-S) interaction and the system has a detection limit of 30 pM, determined by EIS. This was the first aptasensor successfully used to monitor Aβ protein aggregation based on EIS, which was possible because of the aptamer’s high selectivity among Aβ species. With easy fabrication and effective regeneration, this aptasensor might be a promising diagnostic tool for the early detection of AD and for demonstrating Aβ protein accumulation [92].

An aptasensor to detect Aβ oligomers was developed using the α-syn DNA aptamer [91] in complex with methylene blue (MB) and attached to nanoporous anodic alumina. The high absorption coefficient of MB for white light resulted in a low-intensity of reflected white light. In the presence of Aβ oligomers, the aptamer/MB complex dissociated and the increase in reflected light was detected by interferometric reflectance spectroscopy (IRS) [93]. The aptasensor had a detection limit of 20 pM and a good response to the Aβ oligomers, in the concentration range from 0.5 to 50 nM.

Neurofibrillary tangles (NFTs) are pathological hallmarks of AD. The formation of NFTs is initiated in the hippocampus and the extent of NFT formation is associated with the severity of dementia in AD. Hyperphosphorylation of the microtubule-associated protein, tau promotes its ability to recruit and organize normal tau into filaments, which become the NFTs. Thus, hyperphosphorylation of tau is proposed as one of the main causes of AD. In addition to its normal roles in promoting microtubule assembly and stabilizing the assembled microtubules, tau was observed by non-equilibrium capillary electrophoresis (CE) to bind three randomly selected ssDNA oligonucleotides, one of which, ssDNA1, bound to tau381 with high affinity (Kd = 190 nM) [94]. An aptamer/antibody sandwich assay was developed using ssDNA1 to detect femtomolar concentrations of tau protein in human plasma by surface plasmon resonance (SPR) [95]. In another study, DNA aptamers were selected against the entire tau protein of 441 amino acids (tau441) by a rapid selection technique based on capillary electrophoresis partitioning with three selection rounds completed in a single day. Five aptamers chosen from the high throughput sequencing results were evaluated by SPR for their tau recognition ability. The analytical potential of the aptamer with higher affinity was demonstrated by a homogeneous-phase fluorescence anisotropy assay. This high-affinity aptamer bound tau441 protein and the shorter isoforms, tau352, tau381, and tau383 with detection limits of 28 nM, 6.3 nM, 3.2 nM, and 22 nM, respectively, in this assay [96].

#### 3.1.2. AD Therapeutics

As one of the potential therapies, RNA aptamers were selected to inhibit Aβ40 aggregation. During selection, an Aβ oligomer was conjugated to gold nanoparticles (Aβ-AuNPs) and two aptamers were identified as aptamer candidates after SELEX. The Kds of the fluorescently labeled RNAs to monomeric Aβ40 peptide were estimated in the range of 10–20 nM. These aptamers inhibited Aβ plaque and fibril formation as revealed by transmission electron microscopy and an Aβ40 enzyme-linked immunosorbent assay (ELISA) [97]. Alcohol dehydrogenase (ABAD) decoy peptide (DP), which interacts with Aβ, antagonizes the cytotoxicity of the Aβ peptide. The ABAD-DP sequence was inserted between two thiols that form a disulfide bond in thioredoxin (TRX) to create the TRX1-ABAD-DP-TRX2 peptide aptamer. Its stable expression in NIH 3T3 cells using adeno-associated viruses was verified with immunofluorescent staining. The expressed aptamer could bind the Aβ peptide and improve cell viability. This investigation confirmed the effective inhibition of the cytotoxic effect of Aβ peptide by peptide aptamers [98]. Tau proteins normally associate with and stabilize microtubules, but their hyperphosphorylation can result in protein aggregates termed “tauopathy”, which is toxic. DNA aptamer Tau-1, which was selected against the human tau441, inhibits Tau1 oligomerization in vitro [99]. This inhibition was also validated in cell culture studies using HEK293 cells [100].

Beta-secretase (BACE1) has a role in Aβ production, and inhibition of BACE1 expression is lethal in mice. Treatment for AD might involve BACE1 modulation. However, its large active site makes BACE1 a challenging target for small molecule inhibitors. To circumvent this, nucleic acid aptamers might be novel tools to inhibit BACE1 activity. This enzyme has a cytoplasmic tail, B1-CT, serving as a docking site for proteins such as the copper chaperone for superoxide dismutase-1 (CCS) and ADP ribosylation factor-binding (GGA1) protein. The physiological role of the B1-CT is largely unknown. RNA aptamers targeting the B1-CT bound the membrane-proximal half of the C terminus of BACE1 prevented CCS recruitment while allowing GGA1 binding to BACE1 and regulating BACE1 transport to recycling endosomes [101].

To specifically target BACE1, the DNA aptamers, BI1 and BI2, were generated against BACE1, which inhibited BACE1 but neither alpha- nor gamma-secretase. Using a stably transfected HEK293 cell line, they expressed the amyloid protein precursor (APP) and, using an in-vitro fluorescence resonance energy transfer (FRET) assay, they showed that Aβ level was reduced, and cellular deficiency was rescued in a primary cultured neuronal cell line [102]. The aptamer efficiency was further improved by the addition of cholesteryl tetra ethylene glycol (TEG).

### 3.2. Parkinson’s Disease

Parkinson’s disease (PD) is the second most common neurodegenerative disease, affecting about seven million people globally. While being a progressive disease characterized by motor and nonmotor features, it has significant clinical impacts on patients due to their loss of mobility and muscle control. Loss of striatal dopaminergic neurons and neuronal loss in nondopaminergic areas characterize PD. Loss of neurons is generally associated with characteristic Lewy body formation. Thus, the presence of Lewy bodies containing α-syn oligomers in the brain is considered a possible therapeutic and diagnostic target for PD.

#### 3.2.1. PD Diagnostics

The first aptamer, “M5-15”, selected against α-syn lacked specificity for oligomers and could also bind to α-syn monomers [103]. DNA aptamers, then selected, were shown to interact with α-syn oligomers and not α-syn monomers but did bind Aβ1–40 oligomers. The investigators also explored the aptamer’s specificity for α-syn oligomers over other proteins with structures that are predominantly β-sheet, such as the β-sheet propeller structure of pyrroloquinoline quinine glucose dehydrogenase (PQQH), and demonstrated that the aptamer did not bind PQQH [91].

A variety of aptasensors have been reported to be capable of quantifying α-syn oligomers. A colorimetric aptasensor, employing a DNA aptamer against the α-syn oligomer, was adsorbed to gold-nanoparticles (AuNPs), which prevented salt-induced aggregation of the nanoparticles. Its binding to the α-syn oligomer prevents the absorption of aptamer onto the surface of AuNPs, which allows the aggregation of AuNPs to be triggered in high salt concentrations and results in a color change. This system was reported with an LoD of 10 nM. However, serum (even at 2%) contributed absorbing compounds in the range of color change in the assay and interfered with the salt-dependent aggregation of the aptamer-Au-NPs. Aptasensors using EIS and SPR were more sensitive, with LoDs of 1 pM and 8 pM, respectively [104].

An EIS aptasensor was found highly specific for oligomers over monomers and not affected by the presence of serum [104]. An elaborate design for an electrochemical aptasensor to detect α-syn oligomer involved a hybrid formed by the complementary sequence to an α-syn oligomer aptamer linked to a gold surface through a thiol bond and included terminal deoxynucleotidyl transferase (TdT) and dTTP to extend the 3′ end of the aptamer and its complement with additional polyT. The extended ssDNA bound methylene blue and promoted its association with the gold surface, thereby creating an electrochemical signal. In addition, the system contained exonuclease I (Exo I), which is specific for ssDNA. When the aptamer was removed from the binary complex with its complement by its binding to the α-syn oligomer, the complementary strands were digested by Exo I, preventing the accumulation of methylene blue at the gold surface and thus decreasing the electrochemical signal. The detection limit was 10 pM (S/N  =  3) and recovery of the signal in the presence of 10% and lower concentrations of serum was a very acceptable 95.3–107% [105]. However, its complexity and the incorporation of enzymes make this system less likely to be stable to storage and reproducible in the field.

A combination of capillary electrophoresis-mass spectrometry (CE-MS) with solid-phase extraction (SPE) to pre-concentrate the target with an aptamer-modified microcartridge in a large volume of injected samples decreased the detection limit 100-fold compared to CE-MS systems. In this novel system, the sample is cleaned, and the volume is minimized for enhanced analysis by the use of a microcartridge that selectively retains the target. An aptamer affinity (AA) sorbent in the cartridge was found to be a superior alternative to an antibody-based immunoaffinity (IA) sorbents as the background electrolytes required to rinse the system also denature the antibodies. The development of an AA-SPE-CE-MS was achieved by modifying the microcartridge with the M5-15 DNA aptamer. The system demonstrated an LoD of 2.8 nM and had a linear detection range between 7 and 140 nM of α-syn in the blood sample. Also, identification of free and N-acetylated α-syn proteoforms was possible as a result of the highly accurate mass detection and resolving power of the MS. Finally, the microcartridge was found stable even with acidic BGE and could be used for about 20 analyses with a low chance of erroneous quantification of the target [106].

#### 3.2.2. PD Therapeutics

To relieve the symptoms of Parkinson’s disease (PD), two 58-nucleotide-long DNA aptamers specific for human α-syn were selected with Kd values in the nanomolar range (2.4 nM and 3.1 nM). These aptamers effectively reduced aggregation in vitro and intracellular degradation of the α-syn was enhanced by both aptamers. Mitochondrial dysfunction and cellular defects due to overexpression of this protein were consequently rescued [107]. The same group investigated the in-vivo effect of these aptamers. To move the aptamers through the BBB, they packaged the aptamers into exosomes with surface-expressed neuron-specific rabies viral glycoprotein (RVG) peptides. They observed that α-syn accumulation, neuropathological and behavioral deficits were reduced, and motor impairments were improved in a mouse model [108]. Although this study highlights the therapeutic potential of aptamers, more studies are required to target other PD-related proteins, such as DJ-1, LRRK2, DRP-1 and Rab GTPases, as well as α-syn.

### 3.3. Multiple Sclerosis

Multiple sclerosis (MS) is an autoimmune disease in which the myelin sheaths surrounding the neuronal axons in the brain and spinal cord are destroyed. Disease progression is highly unpredictable and refractive to treatment at later stages. Characteristic pathologies of the disease are abnormal activation of microglia and astrocytes, which results in oligodendrocytes and neuronal cell degeneration. While there is no effective cure or diagnosis of the disease, the number of MS patients in the world is greater than 2.5 million [109]. Specific biomarkers that could identify MS early in its progression are not available, anti-inflammatory therapies have faced failures and they only prevent relapses.

#### 3.3.1. MS Diagnostics

A potential solution to the diagnosis problem came from a study of patients with MS and healthy volunteers as controls using modified DNA-aptamers (SOMAscan^®^) to measure more than 1000 proteins in the CSF. Approximately half of the 431 subjects were used as a training set and the other half as a validation set to test the predictive capability of the identified biomarker clusters. This blinded screen identified astrocyte cluster 8 (SERPINA3, MMP7, CLIC1, and GZMA) and microglial cluster 2 (TNFRSF25 and DSG2) as elevated in MS and significantly correlated with MS severity [110]. The astrocyte cluster 8 proteins overlapped with those reported to be associated with neuro-toxic (A1) astrocytes and were demonstrated to be secreted in vitro by primary peripheral blood mononuclear cells in response to lipopolysaccharide, an inflammatory stimulus.

Being an autoimmune disorder, the presence of autoantibodies responsible for demyelination can also be a basis for the diagnosis of MS. A bioluminescent solid-phase sandwich-type microassay was described for detecting autoantibodies related to MS in human serum. This assay is based on a sandwich between two RNA aptamers specific for myelin basic protein autoantibodies, one aptamer linked to a surface and the other linked to the photoprotein, obelin, as a reporter. Application of the assay included 91 serum samples from MS patients and compared with 86 serum samples from healthy individuals. Based on the receiver–operator curve, with an area under the curve of 0.87, a clinical threshold value was defined as 64% sensitivity and 94% specificity. The negative predictive value (NPV) of 96% and likelihood ratio of 10.96 supports the diagnostic value of this assay for MS testing [111].

#### 3.3.2. MS Therapeutics

It was shown that the binding of natural IgM antibodies to oligodendrocytes promotes remyelination of CNS lesions in the mouse [112]. However, IgM antibodies are limited to use in vivo for treating MS because of their size, complexity, and immunogenicity. Therefore, it is of great interest to discover small molecules that are specific to myelin and have the potential to be used for therapeutic remyelination. For this purpose, a 40-nucleotide DNA aptamer, LJM-3064, was identified. Its peritoneal injection resulted in the promotion of remyelination in CNS lesions in mice. It is important to note that LJM-3064 has a G-quadruplex forming guanosine-rich sequence. In another study, the same LJM-3064 aptamer was functionalized with carboxylic acid and conjugated to the exosome surface’s amine groups. It was shown that this conjugation promotes the proliferation of a type of glial cells, oligodendrocytes, that play an important role in myelin sheath formation. When the cells are administered to female mice, the inflammatory response was suppressed and lesions in the CNS were reduced. The system provides a new approach with an effective clinical reality for managing the severity of MS with the help of LJM-3064 aptamers [113]. Thus, LJM-3604 may be an alternative to monoclonal antibodies for MS treatment [114,115].

### 3.4. Amyotrophic Lateral Sclerosis

Dysregulation or alteration of receptor expression levels have been demonstrated in various diseases. Abnormally activated AMPA receptors, which are associated with amyotrophic lateral sclerosis (ALS), are potential drug candidates for ALS treatment. RNA aptamer AN58, raised against the GluR2Q flip AMPA receptor, competitively inhibited the receptor. Its nanomolar affinity is better than NBQX, one of the current best competitive inhibitors. AN58 demonstrated the highest affinity towards GluR2 and higher selectivity for the GluR4 among all AMPA receptor subunits. In short, AN58 is a potential inhibitor with nM affinity of the GluR2 AMPA receptors [116].

Another characteristic of ALS pathology is the toxic accumulation of the TAR DNA-binding protein 43 (TDP-43). Interaction of TDP-43 with RNA results in the regulation of RNA transcription, splicing, transport, and translation. Zacco et al. demonstrated that native partners of TDP-43 can be used to inhibit its aggregation. This inhibition occurs in a length-dependent manner, such that shorter oligos interfere with the aggregation better than long ones. Their study indicates the possibility of increasing protein solubility using a natural interaction that can be adapted as a new therapeutic approach [117].

### 3.5. Huntington Disease

Huntington’s disease (HD) is an incurable hereditary ND, impairing motor and cognitive functions. Huntingtin (HTT) protein is essential for neuronal development; however, some mutations lead to the development of HD pathology. Increases in the number of CAG repeats result in elongation of a polyglutamine stretch of HTT and convert it to a toxic protein, which causes HD. Similar to AD and PD, inhibition of protein aggregation is a promising strategy to slow or stop HD progression [118]. As mentioned before, aptamers, being low- to nonimmunogenic and nontoxic, emerge as robust candidates to interfere with protein–protein interaction and inhibit toxic protein aggregation in such diseases.

High-affinity RNA aptamers were selected against monomeric mHTT (51Q-HTT) and shown to effectively inhibit its aggregation in vitro. Such inhibition diminished oxidative stress in red blood cells (RBCs) and is associated with reduced leakage of a thioflavin-induced fluorescence from liposomes. The presence of aptamers rescued an endocytotic defect and blocked sequestration of glyceraldehyde-3-phosphate dehydrogenase by aggregated mHTT in a yeast model of Huntington’s. Some of these aptamers did not recognize the nonpathogenic, 20Q-HTT, and some increased the number of mHTT in the soluble fraction of yeast. When coexpressed, two successful aptamers increased the efficiency of inhibiting aggregation and improved cell survival. This study implies that using aptamers might be a viable strategy to slow the course of HD [119].

The long polygutamyl stretches cause slight changes in the HTT 3D structure and changes its activity. Indirect modulation of the affected structure using this elongated region on the protein might provide an alternative approach to treatment. G-quadruplex forming DNA aptamers (MS1 to MS4) that bind mHTT significantly decreased the activation by mHTT of the basal histone H3 lysine 27 trimethylation (H3K27me3) activity of the polycomb repressive complex 2 (PRC2) in neuronal progenitor cells (NPCs) from an individual with HD, but not in NPCs from a healthy individual [120]. With this study, DNA aptamers were successfully applied to preferentially target mHTT and to modulate its activity. These two examples provide novel structure-based approaches to effective treatments of mHTT toxicity.

### 3.6. Prion Disease

Transmissible spongiform encephalopathies (TSEs) are a type of neurodegenerative disorder that affect mammals. The pathologic agent associated with these diseases is a misfolded prion protein (PrP). The molecular mechanism (s) resulting in the structural changes that covert the cellular PrP (PrPC) to a pathogenic conformer (PrPSc) are only partially understood, with the most common hypothesis being that a molecular cofactor, acting as a catalyst, favors the transition from PrPC to PrPSc [121].

Three G-quadruplex-forming aptamer sequences were identified for different forms of PrP [122]. These quadruplexes were reported to have high affinity and specificity toward PrP (Kd: 62 nM–630 nM) and a weak affinity for the PrP-β oligomer that mimics the early stage of PrPSc formation. By various analyses, including ITC, SPR, and CD spectroscopy, a high-affinity binding for PrP was associated with a quadruplex structure and PrP terminal domains were required for aptamer binding. This study also provided evidence for a mutual unwinding of nucleic acid and protein upon their interaction. The quadruplex unwinding-activity of PrP was carried out by the intrinsically unstructured N-terminal domain and the DNAs promoted the unfolding of the PrP structured C-terminal domain.

### 3.7. Brain Tumors

Brain tumors are among the most fatal forms of cancer. For example, about two-thirds of adults diagnosed with glioblastoma (a type of brain cancer) lose their life within two years. Brain tumors are also the most common and lethal of all pediatric solid tumors. Children who survive with these tumors also often suffer from the long-term consequences of the necessary medical interventions, such as surgeries and chemotherapies.

Gliomas (glioblastoma, ependymomas, astrocytomas, and oligodendrogliomas) make up almost 80% of all malignant primary tumors of the brain. Glioblastoma multiforme (GBM) is the most frequently observed type of primary astrocytoma constituting almost 60% of all brain tumors in adults [123]. Gliosarcoma, a variant of GBM, is a highly aggressive malignant form of metastatic brain tumor. Although techniques based on tumor morphology (e.g., MRI, histopathology biopsy) to distinguish gliosarcoma from other GBMs, the early diagnosis of this aggressive disease is still poor, and the survival of patients after diagnosis is generally less than one year. Additionally, conventional treatment methods for GBM, such as radiotherapy, chemotherapy, and their combinations, are not effective. The abnormal activity of tumor cells and their resistance to chemotherapy and radiotherapy are major reasons for the higher fatality rate of GBM [124]. Thus, it would be a significant advance to discover specific molecules targeting gliosarcoma for early diagnosis and for treatments such the inhibition of GBM cell activity and increased radiosensitivity [125].

#### 3.7.1. Brain Imaging

Treatment of brain tumors is challenging for clinicians due to the selective permeability of the BBB [126]. The BBB consists of a layer of specialized endothelial cells that controls molecular passage into the brain tissue and prevents the entry of many molecules to maintain the central nervous system (CNS) at a steady state. A variety of techniques have been developed to penetrate the BBB. Invasive techniques are based on the physical breaching of the BBB using techniques like ultrasound, osmotic, or chemical treatments. Non-invasive techniques, on the other hand, do not disrupt the BBB integrity but use chemicals that are capable of crossing the BBB [127].

Imaging allows the observation of features of the brain without invasive procedures. Newer advances in brain imaging are enabling investigations of the dynamic patterns of connectivity between different regions, which are important to learning and memory. Brain images provide information about brain-related diseases and can be created by various techniques, including magnetic resonance imaging (MRI), functional magnetic resonance imaging (fMRI), and positron emission tomography (PET) [128].

MRI is an effective technique that provides non-invasive three-dimensional images of living organisms. Contrast agents, which are usually based on gadolinium (Gd^3+^)-complexes, are used to provide image contrast. DOPTA-Gd, perhaps the first specific MRI contrast agent, responds to Ca^2+^ with a change in the T1 signal an MRI signal of Gd relaxivity. Although other ion-specific contrast agents have been since developed [129], they are still few in number and there is a need for more contrast agents that enable the imaging of small molecules. The T1 relaxivity of Gd changes with its rotational correlation time, which is a function of the molecular mass of the Gd-linked molecule. Nucleic acid aptamers have the potential to be used for this purpose because hybridization with a complement can be altered by the binding of the aptamer target. As an example, the adenosine aptamer was linked to streptavidin and hybridized with a shorter complementary oligonucleotide to which was linked Gd. In the presence of adenosine, the aptamer refolded around its target and dissociated from the complementary Gd-oligonucleotide. Thus, the Gd shifted from being associated with a large (~70 kDa) molecule to a small (~4 kDa) molecule with a resulting decrease in relaxivity and consequent increase in T1 [130].

*PET* is another highly effective imaging method used in clinical diagnostics as it can provide tomographic resolution at any tissue depth. PET imaging is based on positron-emitting radioisotopes, such as ^13^N, ^18^F, ^11^C, ^64^Cu, ^124^I, and ^68^Ga. The fluorine isotope (^18^F) is often used due to its advantageous half-life of about 110 min, effortless production, clean decay and low emission energy. As for MRI, the most important challenge for PET is to design target-specific imaging agents [128]. For this purpose, it was demonstrated that the thrombin aptamer could be photochemically conjugated with 3-azido-5-nitrobenzyl fluoride ([^18^F]ANBF) [70]. Also, PET imaging based on ^18^F -labeled aptamers has been reported for proteins such as tenascin C [131], the protein tyrosine kinase 7 [132], and the EGF receptor [133].

#### 3.7.2. Diagnosis

The epidermal growth factor receptor variant III (EGFRvIII), which is oncogenic due to its constitutive activation rather than being regulated by the EGFR ligands, increases glioma tumorigenicity and resistance to treatment [134]. The U87-EGFRvIII cells overexpressing EGFRvIII were used to obtain DNA aptamers that changed the rate of cell growth and increased radiosensitivity [135]. The binding ability of the aptamers (U2, U8, U19 and U31) to U87-EGFRvIII was confirmed via flow cytometry and confocal microscopy analysis. The aptamer inhibited tumor cell (U87) proliferation and metastasis and affected signaling events downstream of the EGFR. Also, several variants of this aptamer were developed by a series of modifications. While truncation increased its specificity, the insertion of GC pairs into its hairpin stem provided enhanced thermal stability. Aptamers with pyrene modifications identified with the help of molecular docking increased aptamer’s affinity to target molecules [136]. Moreover, ^118^Re-labelled U2 demonstrated antitumor effect in vivo. These promising results encourage the application of the U2 aptamer as a novel therapeutic agent in targeted drug delivery systems [125].

The majority of current therapeutics have failed due to the low specificity of the therapeutic agent that exhibits adverse effects. The main goal of targeted therapy is to enhance the selectivity of drugs and to reduce “off-target” side effects. One approach to achieving this goal is through drug delivery systems using ligands specific to the disease such as aptamer–drug conjugates (ApDCs) [137]. Prodigiosin produced by the bacterium *Serratia marcescens* is cytotoxic with anti-cancer and anti-malarial features. One of its derivatives, prodigiosin 25-C, has immunosuppressive activity. An aptamer conjugated with prodigiosin specifically targets brain cancer cell surfaces. Using a molecular modeling tool, Ascalaph designer software, the glutamate receptor and various aptamers were allowed to interact in NVT ensemble for the duration of 50 ns and at 310 K. From this simulation study, five candidate aptamers were identified based on their delta intermolecular energy (Δ INME). To confirm the simulation data, these selected aptamers were incubated with brain cancer cells and normal brain cells individually. Finally, the specific binding percentage of each aptamer was calculated for both cell types. It was observed that aptamers 8, 10, 11, 23, and 69 have the ability to target epitopes on all brain cancer cells with high affinity and low Δ INME. In addition, among these, the aptamer 10 was adsorbed by brain cancer cells at high levels and its adsorption by normal cells was dramatically low [138].

As mentioned before, a major limitation for brain disorder treatment is the BBB, which restricts the majority of small molecules from entering the brain. The need for tissue-specific targeting is another limiting factor. Delivering drugs to key diseased areas in the brain is an essential approach for the effective therapy. Transferrin (Tf), present on the endothelial cell membranes of the BBB, enables molecules to cross the BBB [139,140,141]. In a recent study, an aptamer targeting the Tf receptor (TfR) was fused to an aptamer binding to EpCAM (epithelial cell adhesion molecule)-expressing cancer cells [142]. The aptamer conjugate maintained specificity and demonstrated enhanced binding affinity for EpCAM and the TfR. Transcytosis of these aptamers through the BBB was confirmed in vivo following a 1-nmol injection. This study showed that bifunctional aptamer chimeras can overcome the BBB and have a potential to specifically treat brain disorders. Utilizing a similar strategy with particles, mesoporous ruthenium [Ru (bpy) 2 (tip)]^2+^ (RBT) nanoparticles (MRN) were provided a dual-targeting function, which was achieved by the aptamer AS1411 (Apt) and Tf grafted on the MRN surfaces that resulted in high anti-cancer drug-loading capacity [143]. This nanosystem RBT@MRN-SS-Tf/Apt enabled effective BBB penetration by Tf and specific targeting to kill the glioma cells in vitro and in vivo. Moreover, the production of reactive oxygen species (ROS) by [Ru (bpy) 2 (tip)]^2+^ induced apoptosis of glioma cells under laser irradiation, enabling photodynamic therapy (PDT), which has been shown to increase the survival period. These drug delivery approaches demonstrate that targeting the TfR can be a successful means of moving cargo across the BBB and that the aptamer chimeras can be efficiently used for this purpose to treat brain tumors and other brain diseases of the CNS.

Small interfering RNAs (siRNA) have the sequence-specific gene-silencing ability, which makes them alternative therapeutic tools. However, the delivery of siRNA into target cells has been challenging, with numerous aptamer-based siRNA delivery systems being developed for enhancing the efficacy of siRNAs [66]. Specific delivery of STAT3 siRNA to GBM cells was achieved using a chimeric aptamer consisting of a siRNA targeting STAT3 (signal transducer and activator of transcription 3) conjugated with a Gint4. T aptamer targeting the PDGFRβ (platelet derived growth factor receptor). STAT3 is a key regulator of the aggressive mesenchymal glioblastoma subtype. The delivery of the system and the silencing of STAT3 were determined in PDGFR β positive GBM cells. It was also shown that the chimera system reduces cell viability and migration in vitro and inhibits tumor growth and angiogenesis in vivo [144].

Aptamers may be applied for diagnosis, as they specifically bind to a variety of glioblastoma cell lines over other cancer cell lines with Kds in the range of 78–168 nM [63]. A family of DNA aptamers was selected and optimized for binding a gliosarcoma cell line (K308) using cell-SELEX [145]. They have Kd values in the nanomolar range with the highest affinity aptamer (WQY-9) having a Kd of 21 nM. WQY-9 was highly selective for K308 cells and was internalized by K308 at 37 °C. When tested against paraffin-embedded tissue samples, the truncated version of WQY-9 (WQY-9-B) stained 73% (11 of 15) gliosarcoma samples compared with 17% of 12 normal samples. A random DNA sequence stained 13 and 20% of gliosarcoma and normal samples respectively. An RNA aptamer (H02) was also selected by cell-SELEX that binds the alpha-5-beta-1 integrin and can distinguish between glioblastoma cell lines and tissues from patient-derived tumor xenografts in cyto- and histo-fluorescence assays [146]. Thus, there are several aptamers with promise for applications to produce more effective gliosarcoma diagnostic tools. Some of the aptamers are listed in Table 3.

## 4. Conclusions and Future Perspective

Countries with rapidly aging populations will be challenged in the future by an increasing number of people affected by several neurodegenerative diseases. By 2050, over two billion people will be over 60 years old and the number of people over 80 will have tripled, from 137 million today to 425 million. This increase in the number of elderly individuals is expected to be accompanied by a proportional rise in the number of patients affected by neurological diseases. An increased incidence of brain tumors is also expected, both because cancer incidence increases with age and is possibly exacerbated by the diminished efficiency of repair mechanisms in the elderly brain. This rise in patients with neurological diseases and brain tumors could be diminished if factors that make the elderly susceptible to neurological disorders and the mechanisms of protein accumulation, impairment in degradation of aggregates, and neuronal cell death were understood, and appropriate diagnostics and treatments were developed. Therefore, understanding the fundamental bases of neurological diseases and brain tumors and the impact on these with aging will identify means of their prevention or cure and improve the quality of life for many people in old age.

Antibodies are currently a primary means of diagnosis of molecular biomarkers of neurological disease, particularly for protein biomarkers. However, antibodies are expensive to produce, have batch-to-batch variation that requires extensive quality control, and they require refrigerated storage. When used therapeutically, antibodies also must first be “humanized” to avoid immune rejection. Opportunities for diagnosis and therapies can be expanded with the inclusion of aptamers that are specific for disease biomarkers and aptamer constructs that can be used to address therapeutic challenges.

Although aptamers provide advantages and many new opportunities for diagnostic and therapeutic applications, their representation in modern diagnostics and therapeutics is low. Compared with antibodies, aptamers are relatively recently discovered molecules and their development into approved diagnostic and therapeutic agents will take time. In this review, we have summarized the available aptamers used for diagnosis and therapy and listed them with sequences and properties in Table 3. However, there are many more potential aptamer targets with links to neurological diseases that include LRRK2, Parkin, PINK1, DRP-1, DJ-1, UBQLN2, C9orf72, NEK-1, and FAS. With further optimization of their function and standardization of their characterization, the application of aptamers is expected to gain momentum and provide for many new opportunities in diagnostics and therapeutics in the field of neuroscience.

## Figures and Tables

**Table 1:** Summary of Alternative SELEX methods.

SELEX Type	Features	Reference
PhotoSELEX	Light sensitive oligonucleotides are excited by UV and covalently link to their target molecules	[14]
Cell-SELEX	Whole cells are used for the selection of aptamers that bind cell surface targets	[15]
In vivo SELEX	Aptamers are selected from an oligonucleotide pool in living animals	[16]
In silico SELEX	Computer programs are used to predict tertiary structure, affinity, and target interaction of aptamer candidates	[17]
CE-SELEX	Capillary electrophoresis is used to select high-affinity aptamers, which reduces the selection process time from weeks to days	[18]
Spiegelmer Technology	After selection, aptamers are synthesized as unnatural L-oligonucleotides, which are more stable than D-oligonucleotides	[19]
Structure Switching SELEX	The nucleic acid pool has a short, unvaried sequence by which all oligonucleotides can be captured on a complementary sequence. The oligonucleotides are released when they switch structures to bind their target molecule.	[13]
Magnetic-assisted Rapid Aptamer Selection (MARAS)	Magnetic nanoparticle-attached targets are used to capture aptamers in the presence of an externally applied rotating magnetic field with varying frequencies that influence the selected aptamer affinities.	[20]
Artificially Expanded Genetic Information (AEGIS)-SELEX	The AEGIS-SELEX library is composed of oligonucleotides containing natural and non-natural nucleosides. These libraries have higher sequence diversities than libraries of oligonucleotides containing only natural nucleosides.	[21]
Robotic Assisted-SELEX	Robotic platforms perform the selection without any manual intervention. It reduces the selection process to less than 2 days	[22]
RAPID-SELEX	A conventional SELEX protocol, but without amplification. After each round, Kd values are measured, and the enriched aptamers are sent to HTS.	[23]
GO-SELEX	A conventional SELEX protocol with unbound oligos adsorbed by graphene oxide (GO)	[24]
Sol-gel SELEX	The desired aptamer target is immobilized on a microfluidic device	[25]
Conditional SELEX	This method enables the selection of aptamers that only function under the chosen condition such as when they are in the presence of a regulatory molecule	[26]
Tailored SELEX	The library sequences do not have primer complements and SELEX is performed in the absence of primer complements. To amplify the selected oligonucleotides, the primer complements are ligated with primers. This method prevents the primer complements on the oligonucleotides from being part of the selected aptamer structure that binds to target	[19]
SPR-SELEX	The desired target is immobilized on an SPR chip and the oligo pool injected on the biosensor chip for aptamer selection.	[27]
Chimeric SELEX	Two or more libraries are used to isolate functionally different aptamers, which are then fused to create a dual function aptamer.	[28]
FRELEX	Random 8mers are used to capture the aptamers in Phase I and the target molecule is free in solution during Phase II of selection. This method allows for a true free aptamer selection strategy.	[29]

**Table 2:** Possible Aptamer Modifications with their Characteristics.

Feature	Modification	Reference
increases stability and resistance to 3′ exonuclease	3′-3′ and 5′-5′ internucleotide linkage	[33]
resistance to 3′ exonuclease	3′ Biotin Conjugates	[34]
increases nuclease resistance	2′-fluoro (2′-F) Substitution	[35]
	2′-amino (2′NH2) Substitution	[35]
	2′-O-methly (2′-OMe) Substitution	[36]
	Triazole replacement	[37]
	L-DNA	[38]
increases DNA nuclease resistance, destabilizes quadruplexes in aptamer structure	thiophosphoryl modifications	[39]
resistance to renal clearance	5′-End with Cholesterol	[40]
	5′-End with Dialkyl Lipids	[41]
	5′-End with PEGylation	[42]
improving binding affinity and target selectivity	base modifications (SOMAmers)	[43]
	structure-based modifications	[44]

**Table 3:** Aptamers used in neuroscience applications.

Aptamer	Sequence (5′-3′)	Target	Kd	Ref.
BT5.6	GGGGACGTAAATTGGATGTGGCTGCTTATGCTCTACTTG	BoNT-E	53 nM	[47]
M-30	GGTATTGAGGGTCGCATCCCGTGGAAACAGGTTCATTGGGCGCAC TCCGCTTTCTGTAGATGGCTCTAACTCTCCTCT	saxitoxin	128 nM	[73]
α-Tox-T2	AGTTAGGGGCGACATGACCAAACGTT	α-toxin	2.85 nM	[74]
Dopa2	GCCGCGGAAGACGUUGGAAGGAUAGAUACCUACAACGGGGAAUAUAGAGGCCACCACAUAGUGAGGCCCUCCUCCCAAG	dopamine	2.8 μM	[77]
T-SO508	GCCTGTGGTGTTGGGGCGGGTGCG	amyloid beta	68 nM	[91]
T-SO530	GGTGCGGCGGGACTAGTGGGTGTG	amyloid beta	63 nM	[91]
ssDNA1	GCGGAGCGTGGCAGG	Tau381	190 nM	[94]
DNA aptamer	-	Tau441	28 nM	[96]
E2	-	amyloid beta 1–40	10.9 μM	[97]
N2	-	amyloid beta 1–40	21.6 μM	[97]
TH14	CGCAACGCCGGGCCACTACGCGAATGGCAAGCCCGTCGAC	BACE1	280 nM	[101]
S10	GTACACGTCGGCCACCTACGCGAAGTGGAAGCCTCATTTG	BACE1	360 nM	[101]
M5-15	-	α-syn	-	[103]
AN58	-	GluR2	-	[116]
MS1	AGGGGTGGGGAGGGGTGGGGA	huntingtin	-	[120]
MS2	AGGGGTGGGGAGGGGAGGGGA	huntingtin	-	[120]
U2	-	EGFRvIII	6.27 nM	[125]
SLYC3	CACTACAGAGGTTGCGTCTGTCCCACGTTGTCATGGGGGGTTGGCCTG	EpCAM	-	[142]
TEPN	GCGCGGTACCGCGCTAACGGATTCCTTTTCCGT	transferrin receptor	65 nM	[142]

## Data Availability

No new data were created or analyzed in this study. Data sharing is not applicable to this article.

## References

[1] Rangel A.E., Chen Z., Ayele T.M., Heemstra J.M. (2018). In vitro selection of an XNA aptamer capable of small-molecule recognition. Nucleic Acids Res..

[2] Ilgu M., Nilsen-hamilton M. (2016). Aptamers in Analytics. Analyst.

[3] Ellington A.D., Szostak J.W. (1990). In vitro selection of RNA molecules that bind specific ligands. Nature.

[4] Robertson D.L., Joyce G.F. (1990). Selection in vitro of an RNA enzyme that specifically cleaves single-stranded DNA. Nature.

[5] Tuerk C., Gold L. (1990). Systematic evolution of ligands by exponential enrichment: RNA ligands to bacteriophage T4 DNA polymerase. Science.

[6] Blind M., Blank M. (2015). Aptamer Selection Technology and Recent Advances. Mol. Ther. Nucleic Acids.

[7] Kang K.-N.N., Lee Y.-S.S. (2013). RNA aptamers: A review of recent trends and applications. Future Trends Biotechnol..

[8] Munzar J.D., Ng A., Juncker D. (2019). Duplexed aptamers: History, design, theory, and application to biosensing. Chem. Soc. Rev..

[9] Zhang Y., Lai B.S., Juhas M. (2019). Recent advances in aptamer discovery and applications. Molecules.

[10] Zhou J., Rossi J. (2017). Aptamers as targeted therapeutics: Current potential and challenges. Nat. Rev. Drug Discov..

[11] Boussebayle A., Groher F., Suess B. (2019). RNA-based Capture-SELEX for the selection of small molecule-binding aptamers. Methods.

[12] Hamaguchi N., Ellington A., Stanton M. (2001). Aptamer beacons for the direct detection of proteins. Anal. Biochem..

[13] Oh S.S., Plakos K., Lou X., Xiao Y., Soh H.T. (2010). In vitro selection of structure-switching, self-reporting aptamers. Proc. Natl. Acad. Sci. USA.

[14] Golden M.C., Collins B.D., Willis M.C., Koch T.H. (2000). Diagnostic potential of PhotoSELEX-evolved ssDNA aptamers. J. Biotechnol..

[15] Ohuchi S. (2012). Cell-Selex technology. BioRes. Open Access.

[16] Sola M., Menon A.P., Moreno B., Meraviglia-Crivelli D., Soldevilla M.M., Cartón-García F., Pastor F. (2020). Aptamers Against Live Targets: Is In Vivo SELEX Finally Coming to the Edge?. Mol. Ther. Nucleic Acids.

[17] Wondergem J.A.J., Schiessel H., Tompitak M. (2017). Performing SELEX experiments in silico. J. Chem. Phys..

[18] Mosing R.K., Bowser M.T. (2009). Isolating aptamers using capillary electrophoresis-SELEX (CE-SELEX). Methods Mol. Biol..

[19] Vater A., Jarosch F., Buchner K., Klussmann S. (2003). Short bioactive Spiegelmers to migraine-associated calcitonin gene-related peptide rapidly identified by a novel approach: Tailored-SELEX. Nucleic Acids Res..

[20] Lai J.C., Hong C.Y. (2014). Magnetic-assisted rapid aptamer selection (MARAS) for generating high-affinity DNA aptamer using rotating magnetic fields. ACS Comb. Sci..

[21] Biondi E., Benner S.A. (2018). Artificially expanded genetic information systems for new aptamer technologies. Biomedicines.

[22] Breuers S., Bryant L.L., Legen T., Mayer G. (2019). Robotic assisted generation of 2′-deoxy-2′-fluoro-modifed RNA aptamers—High performance enabling strategies in aptamer selection. Methods.

[23] Szeto K., Latulippe D.R., Ozer A., Pagano J.M., White B.S., Shalloway D., Lis J.T., Craighead H.G. (2013). RAPID-SELEX for RNA aptamers. PLoS ONE.

[24] Nguyen V.T., Kwon Y.S., Kim J.H., Gu M.B. (2014). Multiple GO-SELEX for efficient screening of flexible aptamers. Chem. Commun..

[25] Ahn J.Y., Jo M., Dua P., Lee D.K., Kim S. (2011). A sol-gel-based microfluidics system enhances the efficiency of RNA aptamer selection. Oligonucleotides.

[26] Smith J.D., Gold L. (2002). Conditional-Selex. U.S. Patent.

[27] Dausse E., Barré A., Aimé A., Groppi A., Rico A., Ainali C., Salgado G., Palau W., Daguerre E., Nikolski M. (2016). Aptamer selection by direct microfluidic recovery and surface plasmon resonance evaluation. Biosens. Bioelectron..

[28] Burke D.H., Willis J.H. (1998). Recombination, RNA evolution, and bifunctional RNA molecules isolated through chimeric SELEX. RNA.

[29] Lecocq S., Spinella K., Dubois B., Lista S., Hampel H., Penner G. (2018). Aptamers as biomarkers for neurological disorders. PLoS ONE.

[30] Tombelli S., Minunni M., Mascini M. (2005). Analytical applications of aptamers. Biosens. Bioelectron..

[31] Hoinka J., Zotenko E., Friedman A., Sauna Z.E., Przytycka T.M. (2012). Identification of sequence-structure RNA binding motifs for SELEX-derived aptamers. Bioinformatics.

[32] Hoinka J., Berezhnoy A., Sauna Z.E., Gilboa E., Przytycka T.M. (2014). AptaCluster—A method to cluster HT-SELEX aptamer pools and lessons from its application. International Conference on Research in Computational Molecular Biology.

[33] Ortigao J.F.R., Rösch H., Montenarh M., Fröhlich A., Seliger H. (1991). Oligonucleotide Analogs with Terminal 3′,3′- and 5′,5′-Internucleotidic Linkages as Antisense Inhibitors of Viral Replication. Antisense Res. Dev..

[34] Dougan H., Lyster D.M., Vo C.V., Stafford A., Weitz J.I., Hobbs J.B. (2000). Extending the lifetime of anticoagulant oligodeoxynucleotide aptamers in blood. Nucl. Med. Biol..

[35] Padilla R., Sousa R. (1999). Efficient synthesis of nucleic acids heavily modified with non-canonical ribose 2′-groups using a mutant T7 RNA polymerase (RNAP). Nucleic Acids Res..

[36] Ruckman J., Green L.S., Beeson J., Waugh S., Gillette W.L., Henninger D.D., Claesson-Welsh L., Janjić N. (1998). 2′-fluoropyrimidine RNA-based aptamers to the 165-amino acid form of vascular endothelial growth factor (VEGF165): Inhibition of receptor binding and VEGF-induced vascular permeability through interactions requiring the exon 7-encoded domain. J. Biol. Chem..

[37] Saccà B., Lacroix L., Mergny J.L. (2005). The effect of chemical modifications on the thermal stability of different G-quadruplex-forming oligonucleotides. Nucleic Acids Res..

[38] Hoellenriegel J., Zboralski D., Maasch C., Rosin N.Y., Wierda W.G., Keating M.J., Kruschinski A., Burger J.A. (2014). The Spiegelmer NOX-A12, a novel CXCL12 inhibitor, interferes with chronic lymphocytic leukemia cell motility and causes chemosensitization. Blood.

[39] Pozmogova G.E., Zaitseva M.A., Smirnov I.P., Shvachko A.G., Murina M.A., Sergeenko V.I. (2010). Anticoagulant effects of thioanalogs of thrombin-binding DNA-aptamer and their stability in the plasma. Bull. Exp. Biol. Med..

[40] Lee C.H., Lee S.H., Kim J.H., Noh Y.H., Noh G.J., Lee S.W. (2015). Pharmacokinetics of a Cholesterol-conjugated Aptamer Against the Hepatitis C Virus (HCV) NS5B Protein. Mol. Ther. Nucleic Acids.

[41] Willis M.C., Collins B., Zhang T., Green L.S., Sebesta D.P., Bell C., Kellogg E., Gill S.C., Magallanez A., Knauer S. (1998). Liposome-anchored vascular endothelial growth factor aptamers. Bioconjug. Chem..

[42] Da Pieve C., Blackshaw E., Missailidis S., Perkins A.C. (2012). PEGylation and biodistribution of an anti-MUC1 aptamer in MCF-7 tumor-bearing mice. Bioconjug. Chem..

[43] Trinh T., Zhu G., Xiao X., Puszyk W., Sefah K., Wu Q., Tan W., Liu C. (2015). A synthetic aptamer-drug adduct for targeted liver cancer therapy. PLoS ONE.

[44] Kato K., Ikeda H., Miyakawa S., Futakawa S., Nonaka Y., Fujiwara M., Okudaira S., Kano K., Aoki J., Morita J. (2016). Structural basis for specific inhibition of Autotaxin by a DNA aptamer. Nat. Struct. Mol. Biol..

[45] Lim Y.C., Kouzani A.Z., Duan W. (2010). Aptasensors: A review. J. Biomed. Nanotechnol..

[46] Hu M., Zhang K. (2013). The application of aptamers in cancer research: An up-to-date review. Future Oncol..

[47] Ren S., Shin H.S., Gedi V., Dua P., Lee D.K., Kim S. (2017). Selection of DNA Aptamers Against Botulinum Neurotoxin E for Development of Fluorescent Aptasensor. Bull. Korean Chem. Soc..

[48] Kim T.H., Lee S.W. (2021). Aptamers for anti-viral therapeutics and diagnostics. Int. J. Mol. Sci..

[49] Marrazza G. (2017). Aptamer Sensors. Biosensors.

[50] Buglak A.A., Samokhvalov A.V., Zherdev A.V., Dzantiev B.B. (2020). Methods and applications of in silico aptamer design and modeling. Int. J. Mol. Sci..

[51] Morita Y., Leslie M., Kameyama H., Volk D.E., Tanaka T. (2018). Aptamer therapeutics in cancer: Current and future. Cancers.

[52] Song S., Wang L., Li J., Fan C., Zhao J. (2008). Aptamer-based biosensors. TrAC Trends Anal. Chem..

[53] Davis K.A., Abrams B., Lin Y., Jayasena S.D. (1996). Use of a high affinity DNA ligand in flow cytometry. Nucleic Acids Res..

[54] Kaur H., Shorie M. (2019). Nanomaterial based aptasensors for clinical and environmental diagnostic applications. Nanoscale Adv..

[55] Kou X., Zhang X., Shao X., Jiang C., Ning L. (2020). Recent advances in optical aptasensor technology for amplification strategies in cancer diagnostics. Anal. Bioanal. Chem..

[56] Yan S.R., Foroughi M.M., Safaei M., Jahani S., Ebrahimpour N., Borhani F., Rezaei Zade Baravati N., Aramesh-Boroujeni Z., Foong L.K. (2020). A review: Recent advances in ultrasensitive and highly specific recognition aptasensors with various detection strategies. Int. J. Biol. Macromol..

[57] Banerjee J., Nilsen-Hamilton M. (2013). Aptamers: Multifunctional molecules for biomedical research. J. Mol. Med..

[58] Catuogno S., Esposito C.L. (2017). Aptamer cell-based selection: Overview and advances. Biomedicines.

[59] Kaur H., Bruno J.G., Kumar A., Sharma T.K. (2018). Aptamers in the therapeutics and diagnostics pipelines. Theranostics.

[60] Rosenberg J.E., Bambury R.M., Van Allen E.M., Drabkin H.A., Lara P.N., Harzstark A.L., Wagle N., Figlin R.A., Smith G.W., Garraway L.A. (2014). A phase II trial of AS1411 (a novel nucleolin-targeted DNA aptamer) in metastatic renal cell carcinoma. Investig. N. Drugs.

[61] Catuogno S., Esposito C.L., de Franciscis V. (2016). Aptamer-mediated targeted delivery of therapeutics: An update. Pharmaceuticals.

[62] Chandola C., Neerathilingam M. (2020). Aptamers for Targeted Delivery: Current Challenges and Future Opportunities. Role of Novel Drug Delivery Vehicles in Nanobiomedicine.

[63] Bayrac A.T., Sefah K., Parekh P., Bayrac C., Gulbakan B., Oktem H.A., Tan W. (2011). In vitro selection of DNA aptamers to glioblastoma multiforme. ACS Chem. Neurosci..

[64] de Almeida C.E.B., Alves L.N., Rocha H.F., Cabral-Neto J.B., Missailidis S. (2017). Aptamer delivery of siRNA, radiopharmaceutics and chemotherapy agents in cancer. Int. J. Pharm..

[65] McNamara J.O., Andrechek E.R., Wang Y., Viles K.D., Rempel R.E., Gilboa E., Sullenger B.A., Giangrande P.H. (2006). Cell type-specific delivery of siRNAs with aptamer-siRNA chimeras. Nat. Biotechnol..

[66] Sivakumar P., Kim S., Kang H.C., Shim M.S. (2019). Targeted siRNA delivery using aptamer-siRNA chimeras and aptamer-conjugated nanoparticles. Wiley Interdiscip. Rev. Nanomed. Nanobiotechnol..

[67] WHO (2019). Dementia. https://www.who.int/news-room/fact-sheets/detail/dementia.

[68] Bouvier-Müller A., Ducongé F. (2018). Nucleic acid aptamers for neurodegenerative diseases. Biochimie.

[69] Qu J., Yu S., Zheng Y., Zheng Y., Yang H., Zhang J. (2017). Aptamer and its applications in neurodegenerative diseases. Cell. Mol. Life Sci..

[70] Lange C.W., VanBrocklin H.F., Taylor S.E. (2002). Photoconjugation of 3-azido-5-nitrobenzyl-[^18^F]fluoride to an oligonucleotide aptamer. J. Label. Compd. Radiopharm..

[71] Rutkowska M., Płotka-Wasylka J., Majchrzak T., Wojnowski W., Mazur-Marzec H., Namieśnik J. (2019). Recent trends in determination of neurotoxins in aquatic environmental samples. TrAC Trends Anal. Chem..

[72] Eissa S., Siaj M., Zourob M. (2015). Aptamer-based competitive electrochemical biosensor for brevetoxin-2. Biosens. Bioelectron..

[73] Gao S., Zheng X., Wu J. (2017). A biolayer interferometry-based competitive biosensor for rapid and sensitive detection of saxitoxin. Sens. Actuators B Chem..

[74] Dhiman A., Anand A., Malhotra A., Khan E., Santra V., Kumar A., Sharma T.K. (2018). Rational truncation of aptamer for cross-species application to detect krait envenomation. Sci. Rep..

[75] Si B., Song E. (2018). Recent advances in the detection of neurotransmitters. Chemosensors.

[76] Masato A., Plotegher N., Boassa D., Bubacco L. (2019). Impaired dopamine metabolism in Parkinson’s disease pathogenesis. Mol. Neurodegener..

[77] Mannironi C., Di Nardo A., Fruscoloni P., Tocchini-Valentini G.P. (1997). In vitro selection of dopamine RNA ligands. Biochemistry.

[78] Liu S., Xing X., Yu J., Lian W., Li J., Cui M., Huang J. (2012). A novel label-free electrochemical aptasensor based on graphene-polyaniline composite film for dopamine determination. Biosens. Bioelectron..

[79] Wei B., Zhong H., Wang L., Liu Y., Xu Y., Zhang J., Xu C., He L., Wang H. (2019). Facile preparation of a collagen-graphene oxide composite: A sensitive and robust electrochemical aptasensor for determining dopamine in biological samples. Int. J. Biol. Macromol..

[80] Li C., Chen X., Zhang Z., Tang J., Zhang B. (2018). Gold Nanoparticle-DNA conjugates enhanced determination of dopamine by aptamer-based microcantilever array sensor. Sens. Actuators B Chem..

[81] Iván G., Szigeti-Csúcs N., Oláh M., Nagy G.M., Góth M.I. (2005). Treatment of pituitary tumors: Dopamine agonists. Endocrine.

[82] Höglinger G.U., Rizk P., Muriel M.P., Duyckaerts C., Oertel W.H., Caille I., Hirsch E.C. (2004). Dopamine depletion impairs precursor cell proliferation in Parkinson disease. Nat. Neurosci..

[83] Jakel R.J., Maragos W.F. (2000). Neuronal cell death in Huntington’s disease: A potential role for dopamine. Trends Neurosci..

[84] Hussain T., Lokhandwala M.F. (2003). Renal dopamine receptors and hypertension. Exp. Biol. Med..

[85] Chávez J.L., Hagen J.A., Kelley-Loughnane N. (2017). Fast and selective plasmonic serotonin detection with Aptamer-gold nanoparticle conjugates. Sensors.

[86] Zhao C., Cheung K.M., Huang I., Yang H., Nakatsuka N., Liu W., Cao Y., Man T., Weiss P.S., Monbouquette H.G. (2021). Implantable aptamer—Field-effect transistor neuroprobes for in vivo neurotransmitter monitoring. Sci. Adv..

[87] Saraf N., Bosak A., Willenberg A., Das S., Willenberg B.J., Seal S. (2017). Colorimetric detection of epinephrine using an optimized paper-based aptasensor. RSC Adv..

[88] Pollak T.A., Rogers J.P., Nagele R.G., Peakman M., Stone J.M., David A.S., McGuire P. (2019). Antibodies in the diagnosis, prognosis, and prediction of psychotic disorders. Schizophr. Bull..

[89] Mroczko B., Groblewska M., Litman-Zawadzka A. (2019). The role of protein misfolding and tau oligomers (TauOs) in Alzheimer’s disease (AD). Int. J. Mol. Sci..

[90] Rahimi F., Murakami K., Summers J.L., Chen C.H.B., Bitan G. (2009). RNA aptamers generated against oligomeric Aβ40 recognize common amyloid aptatopes with low specificity but high sensitivity. PLoS ONE.

[91] Tsukakoshi K., Abe K., Sode K., Ikebukuro K. (2012). Selection of DNA aptamers that recognize alpha-synuclein oligomers using a competitive screening method. Anal. Chem..

[92] Zhang Y., Figueroa-Miranda G., Lyu Z., Zafiu C., Willbold D., Offenhäusser A., Mayer D. (2019). Monitoring amyloid-Β proteins aggregation based on label-free aptasensor. Sens. Actuators B Chem..

[93] Amouzadeh Tabrizi M., Ferré-Borrull J., Marsal L.F. (2019). Highly sensitive aptasensor based on interferometric reflectance spectroscopy for the determination of amyloid Beta as an Alzheimer’s disease biomarkers using nanoporous anodic alumina. Biosens. Bioelectron..

[94] Krylova S.M., Musheev M., Nutiu R., Li Y., Lee G., Krylov S.N. (2005). Tau protein binds single-stranded DNA sequence specifically—The proof obtained in vitro with non-equilibrium capillary electrophoresis of equilibrium mixtures. FEBS Lett..

[95] Kim S., Wark A.W., Lee H.J. (2016). Femtomolar Detection of Tau Proteins in Undiluted Plasma Using Surface Plasmon Resonance. Anal. Chem..

[96] Lisi S., Fiore E., Scarano S., Pascale E., Boehman Y., Ducongé F., Chierici S., Minunni M., Peyrin E., Ravelet C. (2018). Non-SELEX isolation of DNA aptamers for the homogeneous-phase fluorescence anisotropy sensing of tau Proteins. Anal. Chim. Acta.

[97] Takahashi T., Tada K., Mihara H. (2009). RNA aptamers selected against amyloid β-peptide (Aβ) inhibit the aggregation of Aβ. Mol. Biosyst..

[98] Wang X., Yang Y., Jia M.Y., Ma C., Wang M.Y., Che L.H., Yang Y., Wu J. (2013). The novel amyloid-beta peptide aptamer inhibits intracellular amyloid-beta peptide toxicity. Neural Regen. Res..

[99] Teng I.T., Li X., Yadikar H.A., Yang Z., Li L., Lyu Y., Pan X., Wang K.K., Tan W. (2018). Identification and Characterization of DNA Aptamers Specific for Phosphorylation Epitopes of Tau Protein. J. Am. Chem. Soc..

[100] Kim J.H., Kim E., Choi W.H., Lee J., Lee J.H., Lee H., Kim D.E., Suh Y.H., Lee M.J. (2016). Inhibitory RNA Aptamers of Tau Oligomerization and Their Neuroprotective Roles against Proteotoxic Stress. Mol. Pharm..

[101] Rentmeister A., Bill A., Wahle T., Walter J., Famulok M. (2006). RNA aptamers selectively modulate protein recruitment to the cytoplasmic domain of b -secretase BACE1 in vitro. RNA.

[102] Xiang J., Zhang W., Cai X.F., Cai M., Yu Z.H., Yang F., Zhu W., Li X.T., Wu T., Zhang J.S. (2019). DNA Aptamers Targeting BACE1 Reduce Amyloid Levels and Rescue Neuronal Deficiency in Cultured Cells. Mol. Ther. Nucleic Acids.

[103] Tsukakoshi K., Harada R., Sode K., Ikebukuro K. (2010). Screening of DNA aptamer which binds to α-synuclein. Biotechnol. Lett..

[104] Sun K., Xia N., Zhao L., Liu K., Hou W., Liu L. (2017). Aptasensors for the selective detection of alpha-synuclein oligomer by colorimetry, surface plasmon resonance and electrochemical impedance spectroscopy. Sens. Actuators B Chem..

[105] Taghdisi S.M., Danesh N.M., Nameghi M.A., Ramezani M., Alibolandi M., Hassanzadeh-Khayat M., Emrani A.S., Abnous K. (2019). A novel electrochemical aptasensor based on nontarget-induced high accumulation of methylene blue on the surface of electrode for sensing of α-synuclein oligomer. Biosens. Bioelectron..

[106] Pero-Gascon R., Benavente F., Minic Z., Berezovski M.V., Sanz-Nebot V. (2020). On-line Aptamer Affinity Solid-Phase Extraction Capillary Electrophoresis-Mass Spectrometry for the Analysis of Blood α-Synuclein. Anal. Chem..

[107] Zheng Y., Qu J., Xue F., Zheng Y., Yang B., Chang Y., Yang H., Zhang J. (2018). Novel DNA Aptamers for Parkinson’s Disease Treatment Inhibit α-Synuclein Aggregation and Facilitate its Degradation. Mol. Ther. Nucleic Acids.

[108] Ren X., Zhao Y., Xue F., Zheng Y., Huang H., Wang W., Chang Y., Yang H., Zhang J. (2019). Exosomal DNA Aptamer Targeting α-Synuclein Aggregates Reduced Neuropathological Deficits in a Mouse Parkinson’s Disease Model. Mol. Ther. Nucleic Acids.

[109] Wallin M.T., Culpepper W.J., Nichols E., Bhutta Z.A., Gebrehiwot T.T., Hay S.I., Khalil I.A., Krohn K.J., Liang X., Naghavi M. (2019). Global, regional, and national burden of multiple sclerosis 1990–2016: A systematic analysis for the Global Burden of Disease Study 2016. Lancet Neurol..

[110] Masvekar R., Wu T., Kosa P., Barbour C., Fossati V., Bielekova B. (2019). Cerebrospinal fluid biomarkers link toxic astrogliosis and microglial activation to multiple sclerosis severity. Mult. Scler. Relat. Disord..

[111] Krasitskaya V.V., Chaukina V.V., Abroskina M.V., Vorobyeva M.A., Ilminskaya A.A., Kabilov M.R., Prokopenko S.V., Nevinsky G.A., Venyaminova A.G., Frank L.A. (2019). Bioluminescent aptamer-based sandwich-type assay of anti-myelin basic protein autoantibodies associated with multiple sclerosis. Anal. Chim. Acta.

[112] Browne P., Chandraratna D., Angood C., Tremlett H., Baker C., Taylor B.V., Thompson A.J. (2014). Atlas of multiple sclerosis 2013: A growing global problem with widespread inequity. Neurology.

[113] Hosseini Shamili F., Alibolandi M., Rafatpanah H., Abnous K., Mahmoudi M., Kalantari M., Taghdisi S.M., Ramezani M. (2019). Immunomodulatory properties of MSC-derived exosomes armed with high affinity aptamer toward mylein as a platform for reducing multiple sclerosis clinical score. J. Control. Release.

[114] Nastasijevic B., Wright B.R., Smestad J., Warrington A.E., Rodriguez M., Maher L.J. (2012). Remyelination induced by a DNA Aptamer in a mouse model of multiple sclerosis. PLoS ONE.

[115] Voge N.V., Alvarez E. (2019). Monoclonal antibodies in multiple sclerosis: Present and future. Biomedicines.

[116] Huang Z., Pei W., Jayaseelan S., Shi H., Niu L. (2007). RNA Aptamers Selected against the GluR2 Glutamate Receptor Channel. Biochemistry.

[117] Zacco E., Graña-Montes R., Martin S.R., de Groot N.S., Alfano C., Tartaglia G.G., Pastore A. (2019). RNA as a key factor in driving or preventing self-assembly of the TAR DNA-binding protein 43. J. Mol. Biol..

[118] Huang W.J., Chen W.W., Zhang X. (2016). Huntington’s disease: Molecular basis of pathology and status of current therapeutic approaches. Exp. Ther. Med..

[119] Chaudhary R.K., Patel K.A., Patel M.K., Joshi R.H., Roy I. (2015). Inhibition of Aggregation of Mutant Huntingtin by Nucleic Acid Aptamers in Vitro and in a Yeast Model of Huntington’s Disease. Mol. Ther..

[120] Shin B., Jung R., Oh H., Owens G.E., Lee H., Kwak S., Lee R., Cotman S.L., Lee J.M., MacDonald M.E. (2018). Novel DNA Aptamers that Bind to Mutant Huntingtin and Modify Its Activity. Mol. Ther. Nucleic Acids.

[121] Macedo B., Cordeiro Y. (2017). Unraveling prion protein interactions with aptamers and other PrP-binding nucleic acids. Int. J. Mol. Sci..

[122] Cavaliere P., Pagano B., Granata V., Prigent S., Rezaei H., Giancola C., Zagari A. (2013). Cross-talk between prion protein and quadruplex-forming nucleic acids: A dynamic complex formation. Nucleic Acids Res..

[123] Hanif F., Muzaffar K., Perveen K., Malhi S.M., Simjee S.U. (2017). Glioblastoma multiforme: A review of its epidemiology and pathogenesis through clinical presentation and treatment. Asian Pac. J. Cancer Prev..

[124] Bastien J.I.L., McNeill K.A., Fine H.A. (2015). Molecular characterizations of glioblastoma, targeted therapy, and clinical results to date. Cancer.

[125] Zhang X., Peng L., Liang Z., Kou Z., Chen Y., Shi G., Li X., Liang Y., Wang F., Shi Y. (2018). Effects of Aptamer to U87-EGFRvIII Cells on the Proliferation, Radiosensitivity, and Radiotherapy of Glioblastoma Cells. Mol. Ther. Nucleic Acids.

[126] Aldape K., Brindle K.M., Chesler L., Chopra R., Gajjar A., Gilbert M.R., Gottardo N., Gutmann D.H., Hargrave D., Holland E.C. (2019). Challenges to curing primary brain tumours. Nat. Rev. Clin. Oncol..

[127] Bellettato C.M., Scarpa M. (2018). Possible strategies to cross the blood-brain barrier. Ital. J. Pediatr..

[128] Röthlisberger P., Gasse C., Hollenstein M. (2017). Nucleic acid aptamers: Emerging applications in medical imaging, nanotechnology, neurosciences, and drug delivery. Int. J. Mol. Sci..

[129] Xiao Y.D., Paudel R., Liu J., Ma C., Zhang Z.S., Zhou S.K. (2016). MRI contrast agents: Classification and application (Review). Int. J. Mol. Med..

[130] Xu W., Lu Y. (2011). A smart magnetic resonance imaging contrast agent responsive to adenosine based on a DNA aptamer-conjugated gadolinium complex. Chem. Commun..

[131] Jacobson O., Yan X., Niu G., Weiss I.D., Ma Y., Szajek L.P., Shen B., Kiesewetter D.O., Chen X. (2015). PET imaging of tenascin-C with a radiolabeled single-stranded DNA aptamer. J. Nucl. Med..

[132] Jacobson O., Weiss I.D., Wang L., Wang Z., Yang X., Dewhurst A., Ma Y., Zhu G., Niu G., Kiesewetter D.O. (2015). 18F-labeled single-stranded DNA aptamer for PET imaging of protein tyrosine kinase-7 expression. J. Nucl. Med..

[133] Kim H.J., Park J.Y., Lee T.S., Song I.H., Cho Y.L., Chae J.R., Kang H., Lim J.H., Lee J.H., Kang W.J. (2019). PET imaging of HER2 expression with an 18 F-fluoride labeled aptamer. PLoS ONE.

[134] Mukherjee B., McEllin B., Camacho C.V., Tomimatsu N., Sirasanagandala S., Nannepaga S., Hatanpaa K.J., Mickey B., Madden C., Maher E. (2009). EGFRvIII and DNA double-strand break repair: A molecular mechanism for radioresistance in glioblastoma. Cancer Res..

[135] Wu X., Liang H., Tan Y., Yuan C., Li S., Li X., Li G., Shi Y., Zhang X. (2014). Cell-SELEX aptamer for highly specific radionuclide molecular imaging of glioblastoma in vivo. PLoS ONE.

[136] Zavyalova E., Turashev A., Novoseltseva A., Antipova O., Savchenko E., Golovin A., Pavlova G., Kopylov A., Zavyalova E., Novoseltseva A. (2020). Pyrene-Modified DNA Aptamers with High Affinity to Wild-Type EGFR and EGFRvIII. Nucleic Acid Ther..

[137] Zhu G., Niu G., Chen X. (2015). Aptamer-Drug Conjugates. Bioconjug. Chem..

[138] Ayatollahi M., Ayatollahi G., Rashidi M., Hekmatimoghaddam S., Mosshafi M., Jebali A., Iman M., Shahdadi Sardo H. (2018). Prodigiosin-Conjugated Aptamer for Attachment to the Surface of Brain Cancer Cells Mediated by Glutamate Receptor. Colloids Interface Sci. Commun..

[139] Willson J. (2020). Transferrin’ across the blood-brain barrier. Nat. Rev. Drug Discov..

[140] Kariolis M.S., Wells R.C., Getz J.A., Kwan W., Mahon C.S., Tong R., Kim D.J., Srivastava A., Bedard C., Henne K.R. (2020). Brain delivery of therapeutic proteins using an Fc fragment blood-brain barrier transport vehicle in mice and monkeys. Sci. Transl. Med..

[141] Fishman J.B., Rubin J.B., Handrahan J.V., Connor J.R., Fine R.E. (1987). Receptor-mediated transcytosis of transferrin across the blood-brain barrier. J. Neurosci. Res..

[142] Macdonald J., Henri J., Goodman L., Xiang D., Duan W., Shigdar S. (2017). Development of a Bifunctional Aptamer Targeting the Transferrin Receptor and Epithelial Cell Adhesion Molecule (EpCAM) for the Treatment of Brain Cancer Metastases. ACS Chem. Neurosci..

[143] Zhu X., Zhou H., Liu Y., Wen Y., Wei C., Yu Q., Liu J. (2018). Transferrin/aptamer conjugated mesoporous ruthenium nanosystem for redox- controlled and targeted chemo-photodynamic therapy of glioma. Acta Biomater..

[144] Esposito C.L., Nuzzo S., Catuogno S., Romano S., de Nigris F., de Franciscis V. (2018). STAT3 Gene Silencing by Aptamer-siRNA Chimera as Selective Therapeutic for Glioblastoma. Mol. Ther. Nucleic Acids.

[145] Wu Q., Wu L., Wang Y., Zhu Z., Song Y., Tan Y., Wang X.F., Li J., Kang D., Yang C.J. (2016). Evolution of DNA aptamers for malignant brain tumor gliosarcoma cell recognition and clinical tissue imaging. Biosens. Bioelectron..

[146] Fechter P., Cruz Da Silva E., Mercier M.C., Noulet F., Etienne-Seloum N., Guenot D., Lehmann M., Vauchelles R., Martin S., Lelong-Rebel I. (2019). RNA Aptamers Targeting Integrin α5β1 as Probes for Cyto- and Histofluorescence in Glioblastoma. Mol. Ther. Nucleic Acids.

